# Access to RNA-sequencing data from 1,173 plant species: The 1000 Plant transcriptomes initiative (1KP)

**DOI:** 10.1093/gigascience/giz126

**Published:** 2019-10-23

**Authors:** Eric J Carpenter, Naim Matasci, Saravanaraj Ayyampalayam, Shuangxiu Wu, Jing Sun, Jun Yu, Fabio Rocha Jimenez Vieira, Chris Bowler, Richard G Dorrell, Matthew A Gitzendanner, Ling Li, Wensi Du, Kristian K. Ullrich, Norman J Wickett, Todd J Barkmann, Michael S Barker, James H Leebens-Mack, Gane Ka-Shu Wong

**Affiliations:** 1 Department of Biological Sciences, University of Alberta, Edmonton, AB T6G 2E9, Canada; 2 CyVerse, University of Arizona, AZ,1657 East Helen St, Tucson AZ, USA 85721 USA; 4 Georgia Advanced Computing Resource Center, University of Georgia, Athens, GA 30602, US; 5 CAS Key Laboratory of Genome Sciences and Information, Beijing, Institute of Genomics, Chinese Academy of Sciences, Beijing 100101, China; 6 Institut de Biologie de l'ENS (IBENS), Département de biologie, École normale supérieure, CNRS, INSERM, Université PSL, 75005 Paris, France; 7 Department of Biology, University of Florida, Gainesville, FL 32611, USA; 8 BGI-Shenzhen, Beishan Industrial Zone, Yantian District, Shenzhen 518083, China; 9 Department of Evolutionary Genetics, Max Planck Institute for Evolutionary Biology, Plön, Germany; 10 Chicago Botanic Garden, Glencoe, IL 60022; 11 Program in Biological Sciences, Northwestern University, Evanston, IL 60208, USA; 12 Department of Biological Sciences, Western Michigan University, Kalamazoo, MI 49008-5410, USA; 13 Department of Ecology & Evolutionary Biology, University of Arizona, Tucson, AZ 85721, USA; 14 Department of Plant Biology, University of Georgia, Athens, GA 30602, USA; 15 Department of Medicine, University of Alberta, Edmonton, AB T6G 2E1, Canada

**Keywords:** RNA, plants, assemblies, genes, contamination, transcriptome completeness

## Abstract

**Background:**

The 1000 Plant transcriptomes initiative (1KP) explored genetic diversity by sequencing RNA from 1,342 samples representing 1,173 species of green plants (Viridiplantae).

**Findings:**

This data release accompanies the initiative's final/capstone publication on a set of 3 analyses inferring species trees, whole genome duplications, and gene family expansions. These and previous analyses are based on *de novo* transcriptome assemblies and related gene predictions. Here, we assess their data and assembly qualities and explain how we detected potential contaminations.

**Conclusions:**

These data will be useful to plant and/or evolutionary scientists with interests in particular gene families, either across the green plant tree of life or in more focused lineages.

## Data Description

The 1000 Plant transcriptomes initiative (1KP) sequenced and analysed transcribed RNA from 1,342 samples representing 1,173 green plant and chloroplast bearing species, including examples of all major taxa within the Viridiplantae: streptophyte and chlorophyte green algae, bryophytes, ferns, angiosperms, and gymnosperms. Importantly, our selection criteria were not biased towards the model organisms and crop species where other plant sequencing efforts have historically focused. While many of the samples were selected for the phylogenomic analyses, others were motivated by different subprojects.

Major articles describing the project have been published elsewhere [1,2]. The most recent papers [1,3] are focused on large-scale phylogenomic analyses made possible by the breadth of this dataset. While all of the 1,342 samples were used in one analysis or another, published over the course of the last ten years, not all of the samples were judged of adequate quality for every analysis. Since each publication uses different criteria for sample data quality filtering, each necessarily uses a different subset of the sample data; for example the final/capstone paper [1] used only 1,124 species. This Data Note describes the complete dataset and provides additional details on the sample and sequence processing, as well as our quality assessments of these data. It supplements and replaces our earlier work [4] outlining plans for the 1KP efforts.

## Methods

### Sampling strategy

Because of the diversity and the number of species analyzed, no one source could be used. Samples were provided by a global network of collaborators who obtained materials from a variety of sources, including field collection of wild plants, greenhouses, botanical gardens, laboratory specimens, and algal culture collections. To ensure an abundance of expressed genes, we preferred live growing cells, e.g., young leaves, flowers, or shoots, although many samples were also from roots or other tissues. Because of the sample diversity, we did not attempt to define specific standards on growth conditions, time of collection, or age of tissue. For more details, see the supplemental methods in the major analysis article [1].

### RNA extraction

Given the biochemical diversity of these samples, no one RNA extraction protocol was appropriate for all samples. Most samples were extracted using commonly known protocols or using commercial kits. For complete details of the many specific protocols used, please see Appendix S1 of Johnson et al. [5] and Jordon-Thaden et al. [6]. The individual protocols are also available via a protocols.io collection [7]. Depending on the sample, RNA extractions might have been done by the sample provider, a collaborator near the provider, or the sequencing laboratory (BGI-Shenzhen).

### Sequencing at BGI

Samples of extracted RNA or frozen tissues were sent to the sequencing laboratory, BGI-Shenzhen. Prior to library construction, RNA samples were screened by Agilent Bioanalyzer RNA Integrity Number (RIN) scores [8] and basic photometry; obvious low-quality outliers (e.g., RIN scores <6 and/or loss of distinct electropherogram peaks) were excluded. Libraries for Illumina sequencing were constructed using Illumina's standard procedures. Some samples for which only a small amount of RNA was available were processed using TruSeq kits.

Initially, sequencing was done on the Illumina GAII platform, but later samples were run on the HiSeq platform. Associated with this change was a shift from ∼72-bp read lengths to ∼90-bp read lengths (both cases paired-end). Libraries were indexed and multiplexed in the sequencer lanes to a target sequencing depth of 2 Gb per sample. The mean depth achieved was 1.99 Gb of sequence with Phred quality 30 (1 error per thousand bases) or better, and varied across samples, with half of samples in the 1.9–2.5 Gb range as summarized by Table 1.

**Table 1:** Distribution in amount of sequence data per sample library

Percentile	Dataset size (all base qualities) (Gb)
5th	1.3
25th	1.9
50th	2.2
75th	2.5
95th	3.0

Summary percentiles characterizing the sizes of the datasets in gigabase pairs of sequence.

The data were cleaned by eliminating reads containing adapter-primer sequences or high numbers of low-quality bases (i.e., more than half of Phred quality <5 [32% error rate] or >10% uncalled). Sequencing and transcriptome assembly protocols are available in protocols.io [9].

#### *De novo* assembly

Once the data were transferred from BGI, the FastQ files were given a uniform name based on a quasi-random 4-letter identification code. A list of all the samples and their ID code is included in the associated data. These identifiers also distinguish otherwise identical repeated samples and provide a stable reference when a sample's species identification is changed.

Quality filtered reads were assembled using the SOAPdenovo-Trans transcript assembler (version 2012–04-05) [10]. No additional pre-processing of the data was performed. This largely used the program defaults, with the slight modification of increasing the *k*-mer length to 25 bp and reducing the number of processor threads to 1. This reduced thread count allowed us to more efficiently use our computer resources. Both the internal FillGap module and the external GapCloser post-processor (supplied with SOAPdenovo-Trans) were run. An example of the commands used for one of the assemblies (dataset AEPI, *Lineum leonii*):

SOAPdenovo-Trans-31kmer all -s config -p 1 -K 25 -e 2 -F -L 100 -t 5 -o AEPI

GapCloser -a AEPI.scafSeq -b config -o AEPI.GapCloser.fa -l 100 -p 25 -t 1

These commands refer to a configuration file named config, which specified the expected insert size, maximum read length, and read-sequence filenames. The contents of this file were as follows:

max_rd_len = 120

[LIB]

avg_ins = 200

rank = 1

q1 = AEPI-read_1.fq

q2 = AEPI-read_2.fq

When multiple samples from the same species were co-assembled, the last 5 lines were repeated for each data source with the appropriate filenames. Such assemblies were also assigned unique 4-letter identifiers. After assembly, the output contig/scaffold names were modified to create a more informative name containing the assembly's 4-letter identifier, a number within the assembly, and a descriptive name for the species (with additional description of the tissue or other identifier when multiple samples of the same species were sequenced).

### Identification of coding regions and protein translation

To identify likely proteins within the assembled transcripts, sequences were passed through TransPipe [11], which identified reading frames and protein translations by comparison to protein sequences from 22 sequenced and annotated plant genomes in Phytozome (RRID:SCR_006507) [12]. Using BLASTX (RRID:SCR_001653) [13], best-hit proteins were paired with each assembled scaffold at a threshold of 1E−10 expectation value and a minimum length of 100 amino acid residues. Scaffolds that did not have a best-hit protein at this level were removed. These removed scaffolds are predominantly from the numerous short and likely fragmentary sequences; however, some complete genes will have been lost. To determine reading frames and estimate amino acid sequences, each gene is aligned against its best-hit protein by Genewise 2.2.0 (RRID:SCR_015054) [14]. Using the highest-scoring Genewise DNA protein alignments, stop codons and those codons containing ambiguous nucleotides were removed to produce an amino acid sequence for each gene. Outputs in the associated data are paired DNA and protein sequences.

### BLAST searches

Thanks to the support of China National GeneBank (CNGB), a BLAST search service [15] allows public searches against the assemblies and protein translations. CNGB developed the service using NCBI BLAST+ (version 2.6.0) [16]. It integrates all public datasets from CNGB applications, BGI projects, and external data sources and provides a comprehensive and convenient sequence searching. A specialized interface for BLAST searching the 1KP dataset allows limiting the search to specific families, orders, or 25 higher-level clades. For assemblies, there are 21,398,790 nucleotide sequences, 6,188,419,272 bases in total. And for the Transpipe protein translations, there are 103 million protein sequences comprising more than 47 billion amino acids in total.

## Validation

### Purity and contamination

High-throughput sequencing methods are always at risk of contamination [17]. In 1KP, the diversity of sources for the samples, and especially the fact that axenic cultures are not a viable option in most instances, ensure that there will always be some contamination of the plant tissue by other environmental nucleic acids. These can reasonably be expected to include bacterial, fungal, and insect species that live in and on the plant tissues and, more rarely, results of contact with larger species such as frogs, mice, birds, and humans.

For most analyses, these minor contaminants are not expected to matter because only the most abundant of such contaminants will be present in sufficient quantities to assemble. In many cases, they are also sufficiently diverged from the intended species that they can be easily recognized as non-plant genes. Unfortunately, this is not always the case. Some analyses are further protected by looking at the whole of the available transcriptome, whereby the many genes from the target species will overpower a few contaminants. Single gene family analyses do not have this advantage and must rely on other methods to reject non-plant genes.

Another possibility is significant contamination during sample processing when plant RNA is transferred between adjacent samples, or when whole samples are accidentally mislabelled.

Given the potential contamination problems, we tried to identify them in the sequence data by comparing the assembled sequences by BLASTn to a reference set of nuclear 18S ribosomal RNA (rRNA) sequences from the SILVA small subunit (SSU) rRNA database [18,19]. The BLASTn alignment to an assembly with the lowest expectation value is taken to indicate that the assembly has a taxonomic origin similar to that of the reference sequence. However, alignments of under 300 bp or expectation values greater than 1E−9 often align to several distantly related species and were ignored.

For most samples, we found an 18S sequence most similar to a SILVA sequence from the same taxonomic family as the expected sample species. This is not true for all our samples and may indicate a failure to assemble the 18S sequence, limitations in the taxonomic identification from the BLASTn results, or mislabelling of the sample. In a few cases, additional (and possibly contaminant) 18S sequences were found. Because the 18S rRNA sequence is highly expressed, we expect that this method is likely to be sensitive to low levels of contamination. In a few cases, the taxonomic irregularities were judged sufficiently severe that samples were excluded from various analyses.

The accompanying data include 2 accessory files containing details of this SILVA-based SSU validation for each sample [20]. The first lists whether the sample is overall judged to be validated as containing the expected taxon and whether it had alignments to any other plant sequences (described as “worrisome contamination”). The second file, more detailed, lists each scaffold identified as being 18S-like sequence, and which reference sequence it matched against.

It must be emphasized, however, that these files (and indeed this entire section) describe how we removed contaminations from the final analyses. Every publication did their best to removed potential contamination or otherwise inadequate datasets. For example, the final/capstone publication [1] used a subset of only 1,124 species.

### Pairwise cross-contamination of assemblies

Cross-contamination between datasets was also identified by a genome-scale sequence search pipeline, adapted from previous studies [21–23]. Briefly, each pair of assemblies (nucleotide) was compared and a threshold identity level established, above which sequences are likely to be contamination between the pair. While best for identifying technical contamination between libraries (e.g., due to mixing of RNA samples), this technique could also detect other biological contamination events (e.g., contamination of pairs of libraries with common commensal organisms). An additional search step, using the entire 1KP sequence library, identified the probable origin of each sequence.

The pairwise comparison used LAST v. 963 (RRID:SCR_006119) [24] with the –cR01 option, and the respective matches were grouped and ordered by similarity. To avoid artifactually excluding sequences between closely related species, which may have very high degrees of similarity [17], pairs of libraries from the same family, along with pairs of libraries separated by 2 or fewer branches in the consensus 1KP multigene phylogeny, were excluded from the searches [2].

The expected distribution of the matched sequence identities has a maximum at the pairwise identity reflecting the evolutionary distance between the 2 species [22,23]. In contrast, a cross-contaminated pair should contain many sequences of near 100% similarity, and the similarity value that has the first minimum number of sequences below this level (i.e., the first inflection point in a curve plotting the total number of sequences of each percentage similarity value) can be used as a threshold for discriminating contamination sequences [22,23]. This code is available from GitHub [25].

The output of such an analysis is pairs of apparent orthologs whose sequence similarities are higher than the cut-off in one or both libraries, i.e., potential contamination. To discriminate donors and recipients in each contaminant pair, each of these potential contaminants was searched against all the non-contaminant assemblies by BLASTn, using the option -max_target_seqs 3 [26]. Queries with ≥1 of the 3 best alignments against a sequence from the same family, or from a taxon separated by <2 branches within the 1KP tree [2], were excluded from the list of potential contaminants, whereas sequences that yielded best hits exclusively against more distantly related taxa were verified as potential contaminants. Clean and contaminant FASTA sequence files for each library are available in the accompanying data.

**Figure 1:**
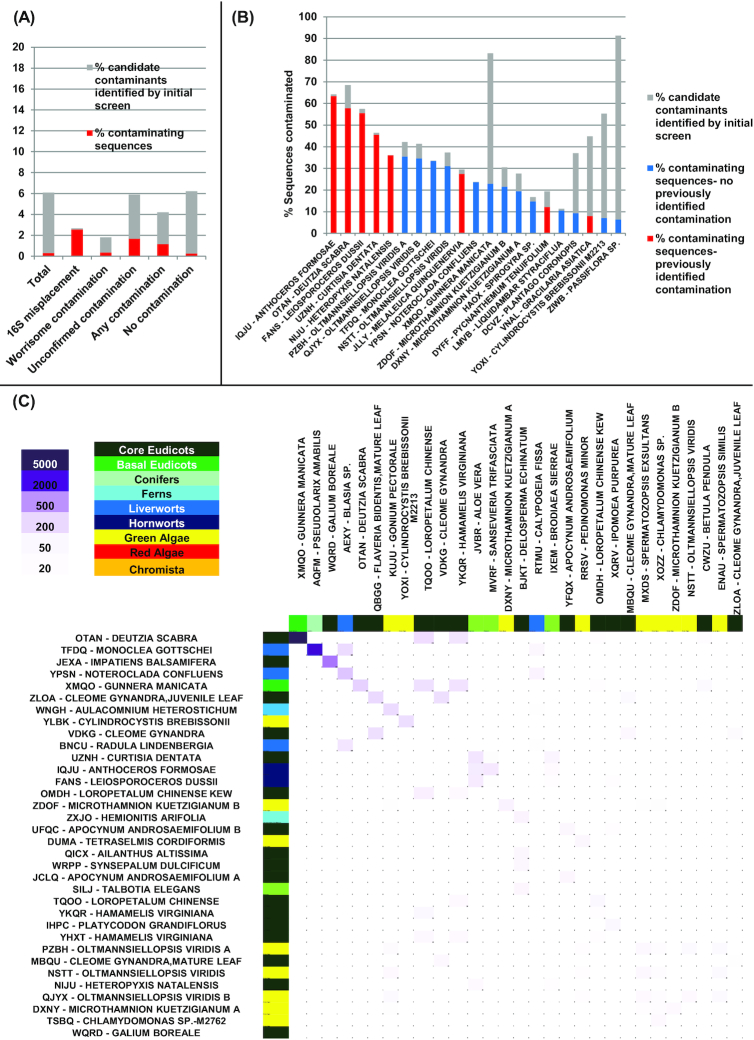
A, Overview of the total sequence percentage verified to be of contaminant origin (red), or inferred to be possible contaminants in other sequence libraries (grey) in all 1KP libraries, and libraries inferred to be contaminated through the 18S phylogenetic placement. B, 21 libraries in which >6% of the total sequences are potential contaminants. C, Heat map of inferred contaminant interactions between pairs of species; contaminated species are shown on the vertical axis and contaminating species on the horizontal axis.

An overview of the results is presented in Fig. 1. In total, we identified 79,175 nucleotide sequences (0.3%) of a total 23,436,405 searched as being clearly of contaminant origin (Fig. 1A). A further 1,477,637 (6.3%) of the sequences either might occur as contaminants in other libraries or could not clearly be identified as being of vertical origin via the search pipeline used. The results obtained were concordant with our other contamination analyses. For example, libraries known to have aberrant 18S sequences contained a much larger average proportion of contaminant sequences (5,890/217,270 sequences [2.7%]) but contained very few sequences that were identified as contaminants in other libraries (252 sequences [0.1%], Fig.   1A). A similar but smaller enrichment in contaminants was identified in libraries identified through 18S sequences as containing unconfirmed contamination (16,871/912,139 sequences [1.8%]), suggesting that at least some of these libraries are genuinely biologically contaminated (Fig. 1A).

Specific libraries contained a much larger proportion of contaminant sequences, with 57.8% of the *Deutzia scabra*(OTAN) sample found to be contaminant (Fig.   1B). These specific contaminations are from *Gunnera manicata* (XMQO) (Fig. 1C), in line with the 18S-based finding. Other cross-contamination events found by this method include *Pseudolarix amabilis* found in *Monoclea gottschei* and *Galium boreale* in *Impatiens balsamifera*. We also, however, identified examples of widespread contamination in libraries that had previously not been detected, e.g., >35% of the sequences detected in 2 libraries of the green alga *Olltmansiellopsis viridis* (Fig. 1B). These may relate to contaminants that do not produce 18S sequences, as evidenced by the recent detection of Rhodobacteralean commensal sequences in 1KP libraries from *Mantoniella squamata* (QXSZ), *Bathycoccus prasinos* (MCPK), and *Nannochloropsis oculata* (JCFK) [27]. Additional results are provided in the associated data release [20].

### Assembly qualities

We assessed the quality of each assembled scaffold/contig using the read-mapping mode of Transrate [28], which detects several classes of common assembly errors and assigns a quality score to each scaffold. Users of the data may choose to omit those portions of the assembly judged as low quality when doing their own analysis. While the assemblies for each sample vary in assessed quality (Table   2), there are thousands of good scaffolds in even the worst of them.

**Table 2:** Assembly quality assessment by Transrate

Percentile	Good scaffolds (all sizes)	Good scaffolds (%)
5th	19,355	32.47
25th	30,755	44.83
50th	37,983	53.65
75th	47,608	62.93
95th	71,368	74.87

Characteristic percentiles summarizing the per sample distributions of high-quality scaffolds for both total counts and fractions of the sample.

**Table 3:** Completeness of gene sets: characteristic percentiles summarizing the distributions of the CEGMA 248 and BUSCO genome completeness scores

Percentile	CEGMA 248	BUSCO*	
		Embryophyta	Eukaryota
5th	79.03	11.2 (8.5)	66.0 (37.3)
25th	89.92	44.1 (29.8)	84.9 (64.4)
50th	92.34	62.5 (48.2)	90.4 (75.9)
75th	93.55	75.2 (59.6)	93.7 (84.1)
95th	94.76	82.6 (73.2)	96.1 (91.0)

^*^BUSCO numbers are the sum of the complete and fragment assembly counts reported, with numbers based on the complete sequence numbers alone given in parentheses.

### Completeness of gene set

Two different approaches were used to estimate transcriptome completeness. First, BUSCO v1 [29] was applied with default settings, using the eukaryote and embryophyte conserved gene datasets (eukaryota_odb9, embryophyta_odb9) as the query databases. Second, conditional reciprocal best BLAST (CRBB) hits were calculated using CRB-BLAST [30] with default parameters. The predicted coding sequences were used as queries against the set of 248 core eukaryotic genes (CEGs) distributed with the CEGMA software; these 248 genes are highly conserved in eukaryotic genomes [31] and hence should be present in most transcriptomes.

**Figure 2:**
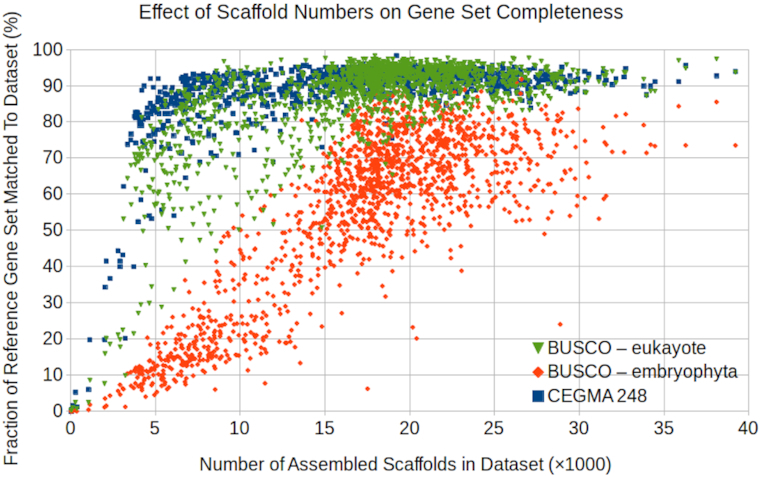
Fraction of the gene sets found (complete + fragments) vs the number of scaffolds (≥300 bp) in the assemblies. For each sample, the fractions of the eukaryota and embryophyta sets found in the assemblies are calculated with BUSCO and the fraction of the CEGMA 248 set with the CRBB tool. All 3 sets are more completely recovered at higher scaffold counts, but the BUSCO embryophyta set is less complete in our samples.

As with all RNA-sequencing data, some genes are more highly expressed than others. While the CEGMA and BUSCO gene sets are intended to demonstrate the completeness of the transcriptomes, they are sensitive to the expression of these genes. Not all these genes will be expressed in the sample's tissues at sufficiently high levels to be assembled. A plot of the number of assembled scaffolds vs the fraction of the 3 gene sets found in the assembled scaffolds shows an increase in the gene fractions found as the number of assembled scaffolds increases (Fig. 2). However, these quickly saturate at ≥80% for the CEGMA and BUSCO-eukaryote sets, with a continuing increase over a larger range for the BUSCO-embryophyte set.

This shows that the 3 gene sets have somewhat different expression patterns, with the CEGMA and BUSCO-eukaryotic sets comprising genes that are more readily detected in our RNA samples. Some of the weaker sensitivity to the BUSCO-embryophyte set is attributable to our sampling species outside of this phylum, which may not have the homologous genes; however, the difference is present when only the embryophyte samples are considered (not shown).

Percentage CEG abundance was calculated as number of CEGs with a CRBB hit divided by 248, the number of CEGs used. The percentage BUSCO abundance was calculated as 100% minus the missing percentage. Samples with low abundance by these measures should be treated with caution because the observed transcriptome incompleteness may indicate problems in library preparation or other types of poor sample quality. For these reasons the taxonomic analyses in [1] excluded samples with <57.5% BUSCO abundance. Table 3 shows the percentages of complete genes found for each of the 3 references at several percentile levels of the whole dataset.

### Re-use potential

Because many of the samples are from poorly studied taxa, these are the first-large scale sequence data to be made available for many species. We expect these sequences to be of broad interest to the plant sciences community, whether researchers merely use our sequences, supplement them with their own sequences, or develop PCR primer and probe sets to collect entirely new sequence data.

## Availability of Supporting Data and Materials

All sequencing read data are available as EBI BioProject PRJEB4922. Data and results of analyses from the final/capstone publication [1] are available in Cyverse Data Commons [32]. All other supporting data presented here are associated with a GigaDB submission [20]. The GigaDB materials include:

1. Tables with list of samples/assemblies and corresponding ENA/NCBI references and GigaDB links.
2. The major part of the provided data have a directory for each assembly. This is named based on the 4-letter code and a species name. Within the directory are a FASTA file containing the SOAPdenovo-Trans assembly, translations of the scaffolds to amino acids, the subset of the nucleotide sequence corresponding to the translation, and tab-separated (text) files with tables of Transrate outputs assessing the assemblies and lists of the reference sequence that each translation is based on. These are available for each of the assemblies.
3. Two accessory tables containing details of the SILVA-based SSU validation for each sample. One file lists whether the sample is overall judged to be validated as containing the expected sequence and whether it had alignments to any other plant sequences (described as worrisome contamination). The second file has more details listing each scaffold identified as being an 18S sequence, and which reference sequence it matched against.
4. The cross-contamination details. One summary file includes a table with the number of contaminants, number of non-contaminant sequences, and the number of sequences inferred to be contaminants in other taxa for each sequence library. Also included is a list of each pair of contaminant sequences identified, with the first column showing the contaminant sequence, and the second column the sequence corresponding to the orthologous contaminating partner against which the sequence was identified. Also included is a list of taxonomically close sample pairs that were not compared. Clean and contaminant FASTA sequence files for each library are also available in the accompanying data.

## Availability of Supporting Source Code and Requirements

- Project name: Decontamination-pipeline
- Project home page: https://github.com/Plant-and-diatom-genomics-IBENS-Paris/Decontamination-pipeline
- Operating system: Linux
- Programming language: Bash
- Other requirements: LAST, join C++ libraries
- License: GNU GPL v3

## Abbreviations

1KP: 1000 Plant transcriptomes initiative; BLAST: Basic Local Alignment Search Tool; bp: base pairs; BUSCO: Benchmarking Universal Single-Copy Orthologs; CEG: Core Eukaryotic Gene; CEGMA: Core Eukaryotic Genes Mapping Approach; CNGB: China National GeneBank; CRBB: Conditional Reciprocal Best BLAST; Gb: gigabase; ENA: European Nucleotide Archive; NCBI: National Center for Biotechnology Information; RIN: RNA Integrity Number; rRNA: ribosomal RNA; SSU: Small SubUnit.

## Competing Interests

The authors declare that they have no competing interests, and that they believe that all the plant tissues were collected in accordance with applicable regulations and laws.

## Authors' Contributions

E.J.C., N.M., S.A., S.W., J.S., J.Y., F.R.J.V., C.B., R.G.D., K.U, N.J.W., T.J.B., and M.S.B. performed data analyses; L.L. and W.D. produced the BLAST website; E.J.C., M.A.G., and R.G.D. wrote the manuscript; J.H.L.-M. and G.K.-S.W. managed and supervised the work.

## Supplementary Material

giz126_GIGA-D-19-00241_Original_Submission

giz126_GIGA-D-19-00241_Revision_1

giz126_GIGA-D-19-00241_Revision_2

giz126_Response_to_Reviewer_Comments_Original_Submission

giz126_Response_to_Reviewer_Comments_Revision_1

giz126_Reviewer_1_Report_Original_Submission Yongshuai Sun -- 7/2/2019 Reviewed

giz126_Reviewer_1_Report_Revision_1 Yongshuai Sun -- 8/9/2019 Reviewed

giz126_Reviewer_2_Report_Original_Submission Erik Alexandersson -- 7/6/2019 Reviewed
