# Supplementary material for: Access to RNA-sequencing data from 1,173 plant species: The 1000 Plant transcriptomes initiative (1KP)
Source: Gigascience. 2019 Oct 23;8(10):giz126. doi: 10.1093/gigascience/giz126 (PMC6808545; doi:10.1093/gigascience/giz126)

GigaScience

Data for "A Phylogenomic View of Evolutionary Complexity in Green Plants"

--Manuscript Draft--

**Manuscript Number:**	GIGA-D-19-00241
**Full Title:**	Data for "A Phylogenomic View of Evolutionary Complexity in Green Plants"
**Article Type:**	Data Note
**Funding Information:**	Alberta Innovates - Technology Futures (RES0010334) — Prof Gane Ka-Shu Wong
**Abstract:**	The 1000 Plants (1KP) initiative explored the genetic diversity of green plants (Viridiplantae) by sequencing RNA from 1,342 samples representing 1,173 species. All of the analyses done for the 1KP capstone, and previous studies on subsets of these data, are based on a series of de novo transcriptome assemblies and related outputs that will be described in this publication. We also describe assessments of the data quality and an analysis to remove cross-contamination between the samples. These data will be useful to researchers with interests in specific gene families, either across the green plant tree of life or in more focused lineages.
**Corresponding Author:**	Gane Ka-Shu Wong<br><br>CANADA
**Corresponding Author Secondary Information:**	
**Corresponding Author's Institution:**	
**Corresponding Author's Secondary Institution:**	
**First Author:**	Eric J. Carpenter
**First Author Secondary Information:**	
**Order of Authors:**	Eric J. Carpenter<br>Gane Ka-Shu Wong
**Order of Authors Secondary Information:**	
**Additional Information:**	
**Question**	**Response**
Are you submitting this manuscript to a special series or article collection?	No
**Experimental design and statistics**<br><br>Full details of the experimental design and statistical methods used should be given in the Methods section, as detailed in our Minimum Standards Reporting Checklist. Information essential to interpreting the data presented should be made available in the figure legends.<br><br>Have you included all the information requested in your manuscript?	Yes

Powered by Editorial Manager® and ProduXion Manager® from Aries Systems Corporation

**Resources**<br><br>A description of all resources used, including antibodies, cell lines, animals and software tools, with enough information to allow them to be uniquely identified, should be included in the Methods section. Authors are strongly encouraged to cite Research Resource Identifiers (RRIDs) for antibodies, model organisms and tools, where possible.<br><br>Have you included the information requested as detailed in our Minimum Standards Reporting Checklist?	No
If not, please give reasons for any omissions below.<br><br>as follow-up to "**Resources**<br><br>A description of all resources used, including antibodies, cell lines, animals and software tools, with enough information to allow them to be uniquely identified, should be included in the Methods section. Authors are strongly encouraged to cite Research Resource Identifiers (RRIDs) for antibodies, model organisms and tools, where possible.<br><br>Have you included the information requested as detailed in our Minimum Standards Reporting Checklist?<br><br>"	The data is derived from plant samples for which no attempt was made to identify an age or sex for the source.
**Availability of data and materials**<br><br>All datasets and code on which the conclusions of the paper rely must be either included in your submission or deposited in publicly available repositories (where available and ethically appropriate), referencing such data using	No

Powered by Editorial Manager® and ProduXion Manager® from Aries Systems Corporation

a unique identifier in the references and in the “Availability of Data and Materials” section of your manuscript.<br><br>Have you have met the above requirement as detailed in our Minimum Standards Reporting Checklist?	
If not, please give reasons for any omissions below.<br><br>as follow-up to "**Availability of data and materials**<br><br>All datasets and code on which the conclusions of the paper rely must be either included in your submission or deposited in publicly available repositories (where available and ethically appropriate), referencing such data using a unique identifier in the references and in the “Availability of Data and Materials” section of your manuscript.<br><br>Have you have met the above requirement as detailed in our Minimum Standards Reporting Checklist?<br><br>"	Additional data (contamination analysis, etc) will be submitted to GigaDB after this online process, as per the journal instructions.

Powered by Editorial Manager® and ProduXion Manager® from Aries Systems Corporation

Data for "1 A Phylogenomic View of Evolutionary Complexity in Green Plants" Click here to download Manuscript data release for 1kp-raw-assemblies 20190627.docx

Title: The Data for One Thousand Plant Transcriptomes Initiative: A Phylogenomic View of Evolutionary Complexity in Green Plants

Authors:

Eric J. Carpenter <ejc@ualberta.ca> [1]

Naim Matasci <nmatasci@usc.edu> [2]

Saravanaraj Ayyampalayam <raj@plantbio.uga.edu> [3]

Shuangxiu Wu <wushx@big.ac.cn> [4]

Jing Sun <jsun@genetics.ac.cn> [4]

Jun Yu <junyu@big.ac.cn> [4]

Fabio Rocha Jimenez Vieira <rocha@biologie.ens.fr> [5]

Chris Bowler <cbowler@biologie.ens.fr> [5]

Richard G. Dorrell <dorrell@biologie.ens.fr> [5]

Matthew A. Gitzendanner <magitz@ufl.edu> [6]

Ling Li <liling3@cngb.org> [7]

Wensi Du <duwensi@cngb.org> [7]

Kristian Ullrich <ullrich@evolbio.mpg.de> [8]

Norm J. Wickett <norman.wickett@gmail.com> [9]

Todd J. Barkmann <todd.barkman@wmich.edu> [10]

Michael S. Barker <msbarker@email.arizona.edu> [11]

James H. Leebens-Mack <jleebensmack@uga.edu> [3]

Gane Ka-Shu Wong <gane@ualberta.ca>* contact author [1,7,12]

1. Department of Biological Sciences, University of Alberta, Edmonton, Alberta, T6G 2E9, Canada.

2. CyVerse, University of Arizona, Arizona, U.S.A.; Current address: Lawrence J. Ellison Institute for Transformative Medicine, University of Southern California, Los Angeles, CA 90033, U.S.A.
3. Department of Plant Biology, University of Georgia, Athens, GA 30602, USA.
4. CAS Key Laboratory of Genome Sciences and Information, Beijing, Institute of Genomics, Chinese Academy of Sciences, Beijing 100101, People's Republic of China.
5. Institut de Biologie de l'ENS (IBENS), Département de biologie, École normale supérieure, CNRS, INSERM, Université PSL, 75005 Paris, France
6. Department of Biology, University of Florida, Gainesville, Florida 32611, USA.
7. BGI-Shenzhen, Beishan Industrial Zone, Yantian District, Shenzhen 518083, People's Republic of China.
8. Department of Evolutionary Genetics, Max Planck Institute for Evolutionary Biology, Plön, Germany.
9. Chicago Botanic Garden, Glencoe, IL 60022, and Program in Biological Sciences, Northwestern University, Evanston, IL 60208 USA.
10. Department of Biological Sciences, Western Michigan University, Kalamazoo MI 49008-5410 USA.
11. Department of Ecology & Evolutionary Biology, University of Arizona, Tucson, AZ 85721 USA.
12. Department of Medicine, University of Alberta, Edmonton, Alberta, T6G 2E1, Canada.

Abstract

The 1000 Plants (1KP) initiative explored the genetic diversity of green plants (Viridiplantae) by sequencing RNA from 1,342 samples representing 1,173 species. All of the analyses done for the 1KP capstone, and previous studies on subsets of these data, are based on a series of de novo transcriptome assemblies and related outputs that will be described in this publication. We also describe assessments of the data quality and an analysis to remove cross-contamination between the samples. These data will

be useful to researchers with interests in specific gene families, either across the green plant tree of life or in more focused lineages.

Keywords

RNA, plants, assemblies, genes, contamination, completeness

Data Description

1KP has sequenced RNA from 1,342 RNA samples of 1,173 green plant species representing all major taxa within the Viridiplantae, including streptophyte and chlorophyte green algae, bryophytes, ferns, angiosperms, and gymnosperms. Importantly, our selection criteria eschewed the model organisms and crop species where other plant sequencing efforts have historically been concentrated.

Major papers describing the project have been published elsewhere [1,2]. This Data Note describes the sequence data set and provides additional details on the sample and sequence processing as well as quality assessments of these data.

Methods

Sampling strategy

Because of the diversity and the number of species analyzed, no one source could be used. Samples were provided by a global network of collaborators who obtained materials from a variety of sources, including field collection of wild plants, greenhouses, botanical gardens, laboratory specimens, and algal culture collections. To ensure an abundance of expressed genes, we preferred live growing cells, e.g. young leaves, flowers, or shoots, although many samples were also from roots, or other tissues. Because of the sample diversity, we did not attempt to define specific standards on growth conditions, time of collection, or age of tissue. For more details, see the supplemental methods in the capstone paper [1].

RNA extraction

Given the biochemical diversity of these samples, no one RNA extraction protocol was appropriate for all samples. Most samples were extracted using commonly known protocols or using commercial kits. For complete details of the many specific protocols used, please see Appendix S1 of Johnson et al. [3] and Jordon-Thaden et al. [4]. Depending on the sample, RNA extractions might have been done by the sample provider, a collaborator near the provider, or the sequencing lab (BGI-Shenzhen).

Sequencing at BGI

Samples of extracted RNA or frozen tissues were sent to the sequencing lab, BGI-Shenzhen. Prior to library construction, RNA samples were screened by Agilent Bioanalyzer RIN scores [5] and basic photometry; obvious low-quality outliers (e.g., RIN scores less than 6 and/or loss of distinct electropherogram peaks) were excluded. Libraries for Illumina sequencing were constructed using Illumina's standard procedure. Some samples for which only a small amount of RNA was available were processed using TruSeq kits.

Initially, sequencing was done on the Illumina GAII platform, but later samples were run on the HiSeq platform. Associated with this change was a shift from ~72 bp read lengths to 90 bp read lengths (both cases paired-end). Libraries were indexed and multiplexed in the sequencer lanes to a target sequencing depth of 2 Gbp per sample. Average depth achieved was 1.99 Gbp of sequence of better than Phred quality 30 (1 error per thousand bases).

Percentile	Dataset Size (all base qualities)
5th	1.3 Gbp
25th	1.9 Gbp
50th	2.2 Gbp
75th	2.5 Gbp
95th	3.0 Gbp

The data was cleaned by eliminating reads with excessive adapter-primer sequences or high numbers of low quality bases (i.e. more than half of Phred quality 5 or lower [32 % error rate] or more than 10% uncalled).

De novo assembly

Quality filtered reads were assembled using the SOAPdenovo-Trans transcript assembler (version 2012-04-05) [6]. No additional pre-processing of the data was performed. This largely used the program defaults, with the slight modification of increasing the *k*-mer length to 25 bp and reducing the number of processor threads to one. This reduced thread count allowed us to more efficiently use our

computer resources. Both the internal FillGap module and the external GapCloser post-processor (supplied with SOAPdenovo-Trans) were run. An example of the commands used for one of the assemblies (dataset AEPI):

```
SOAPdenovo-Trans-31kmer all -s config -p 1 -K 25 -e 2 -F -L 100 -t 5 -o AEPI
GapCloser -a AEPI.scafSeq -b config -o AEPI.GapCloser.fa -l 100 -p 25 -t 1
```

These commands refer to a configuration file named config, which specified the expected insert size, maximum read length, and read-sequence filenames. The contents of this file were:

```
max_rd_len=120
[LIB]
avg_ins=200
rank=1
q1=AEPI-read_1.fq
q2=AEPI-read_2.fq
```

When multiple samples from the same species were co-assembled, the last five lines were repeated for each data source with the appropriate filenames. See the supplemental files in the accompanying analysis paper [1] and protocols in protocols.io for more details [cite].

Protein translation

To identify likely proteins within the assembled transcripts, sequences were passed through TransPipe [7], which identified reading frames and protein translations by comparison to protein sequences from 22 sequenced and annotated plant genomes in Phytozome [8]. Using BLASTX [9], best hit proteins

were paired with each assembled scaffold at a threshold of 1E-10 expectation-value and a minimum length of 100 amino acid residues. Scaffolds that did not have a best hit protein at this level were removed. To determine reading frames and estimate amino acid sequences, each gene is aligned against its best hit protein by Genewise 2.2.0 [10]. Using the highest scoring Genewise DNA-protein alignments, stop codons and those containing ambiguous nucleotides were removed to produce an amino acid sequence for each gene. Outputs include paired DNA and protein sequences.

BLAST searches

Thanks to the support of China National GeneBank (CNGB), a BLAST search service (http://db.cngb.org/onekp/) allows public searches against the assemblies and protein translations. CNGB developed the service using NCBI BLAST+ (version 2.6.0) [11]. It integrates all public datasets from CNGB applications, BGI projects and external data sources, and provides a comprehensive and convenient sequence searching. A specialized interface for BLAST searching the 1KP dataset allows limiting the search to specific families, orders, or 25 higher-level clades. For assemblies, there are 21,398,790 nucleotide sequences, 6,188,419,272 bases in total. And for the Transpipe protein translations, there are 103 million protein sequences comprising over 47 billion amino acids in total.

Validation

Purity and contamination

High throughput sequencing methods are always at risk of contamination, as even a 1 ppm contaminant produces multiple reads. In practice, data has been found to often include sequences best attributed to additional contaminating sources [12]. For 1KP, the diversity of sources for the samples, and especially the fact that axenic cultures are not a viable option in most instances, ensures that there will always be some contamination of the plant tissue by other environmental nucleic acids. These can reasonably be expected to include bacterial, fungal, and insect species that live in and on the plant tissues, and more rarely, from contact with larger species such as frogs, mice, birds and humans.

For most analyses, these minor contaminants are not expected to matter, as only the most abundant of such contaminants will be present in sufficient quantities to assemble. In many cases, they are also sufficiently diverged from the intended species that they can be easily recognised as non-plant genes. Unfortunately, this is not always the case. Some analyses are further protected by looking at the whole of the available transcriptome, whereby the many genes from the target species will overpower a few contaminants. Single gene family analyses do not have this advantage and must rely on other methods to reject non-plant genes.

Another possibility is significant contamination during sample processing when plant RNA is transferred between adjacent samples, or when whole samples are accidentally mis-labeled.

We tried to guard against these problems by several analyses, one of which compared the assembled sequences by BLASTn to a reference set of nuclear 18S rRNA sequences from the SILVA SSU rRNA database (http://www.arb-silva.de) [13]. The BLASTn alignment to an assembly with the lowest expectation-value is taken to indicate the assembly has a similar taxonomic origin as the reference sequence. However, alignments of less than 300 bp or expectation-values above 1E-9 often align to several distantly related species and were ignored.

For most samples we found an 18S sequence most-similar to a SILVA sequence from the same taxonomic family as the expected sample species. This is not true for all our samples, and may indicate a failure to assemble the 18S sequence, limitations in the taxonomic identification from the BLASTn results, or mis-labelling of sample. In a few cases, additional (and possibly contaminant) 18S sequences were found. Because the 18S rRNA sequence is highly expressed, we expect that this method is likely to be sensitive to low levels of contamination. In a few cases, the taxonomic irregularities were judged sufficiently severe that samples were excluded from various analyses.

The accompanying data includes two accessory files containing details of this SILVA based SSU validation for each sample. The first lists whether the sample is overall judged to be validated as containing the expected taxon, and whether it had alignments to any other plant sequences (described as "worrisome contamination"). The second file, more detailed, lists each scaffold identified as being 18S-like sequence, and which reference sequence it matched against.

Pairwise Cross-contamination of Assemblies

Cross contamination between the datasets was identified by using a genome-scale sequence search pipeline, adapted from previous studies [14-16]. Briefly, each pair of assemblies (nucleotide) was compared and a threshold identity level established, above which sequences are likely to be contamination between the pair. While best for identifying technical contamination between libraries (e.g. due to mixing of RNA samples), this technique could also detect other biological contamination events (e.g. contamination of pairs of libraries with common commensal organisms). An additional search step, using the entire 1KP sequence library, identified the probable evolutionary origin of each sequences.

The pair-wise comparison used LAST v. 963 [17] using the --cR01 option, and the respective matches were grouped and ordered by similarity. To avoid artifactually excluding sequences between closely related species, which may have very high degrees of similarity [13], pairs of libraries from the same family, along with pairs of libraries separated by two or fewer branches in the consensus 1kp multigene phylogeny, were excluded from the searches [2].

The expected distribution of the matched sequence identities has a maximum at the pairwise identity reflecting the evolutionary distance between the two species [15, 16]. In contrast, a cross-contaminated pair should contain many sequences of near 100% similarity, and the similarity value which has the first minimum number of sequences below this level (i.e. the first inflexion point in a curve plotting the total number of sequences of each percentage similarity value) can be used as a threshold for discriminating contaminating sequences [15, 16]. The code is available at https://github.com/Plant-and-diatom-genomics-IBENS-Paris/Decontamination-pipeline.

The output of this analysis is pairs of apparent orthologs whose sequence similarities are higher than the cut-off in one or both libraries, i.e. potential contamination. To discriminate donors and recipients in each contaminant pair, each of these potential contaminants was searched against all the non-contaminant assemblies by BLASTn, using the option -max_target_seqs 3 [18]. Queries with at least one of the three best alignments against a sequence from the same family, or from a taxon separated by fewer than two branches within the 1kp tree [2], were excluded from the list of potential contaminants; whereas sequences that yielded best hits exclusively against more distantly related taxa, were verified as potential contaminants. Clean and contaminant FASTA sequence files for each library are available in the accompanying data.

An overview of the results is presented in Fig. 1. In total, we identified 79,175 nucleotide sequences (0.3 %) of a total 23,436,405 searched as being clearly of contaminant origin (Fig. 1A). A further

1,477,637 (6.3%) of the sequences might either occur as contaminants in other libraries, or could not clearly be identified as being of vertical origin via the search pipeline used. The results obtained were concordant with the other contamination analyses. For example, libraries known to have aberrant 18S sequences contained a much larger average proportion of contaminant sequences (5.890/217,270 sequences, 2.7 %), but contained very few sequences that were identified as contaminants in other libraries (252 sequences, 0.1%, Fig. 1A). A similar, but smaller enrichment in contaminants was identified in libraries identified through 18S sequences as containing unconfirmed contamination (16,871/ 912139 sequences; 1.8%), suggesting that at least some of these libraries are genuinely biologically contaminated (Fig. 1A).

Specific libraries contained a much larger proportion of contaminant sequences, with 57.8% of the *Deutzia scabia* (OTAN) found to be contaminant (Fig. 1B). These specific contaminations are from *Gunnera manicata* (XMQO) (Fig. 1C), in line with the 18S based finding. Other cross-contamination events found by this method include *Pseudolarix amabilis* found in *Monoclea gottschei* and *Galium boreale* in *Impatien balsamifera*. We also, however, identified examples of widespread contamination in libraries that had previously not been detected, for example over 35% of the sequences detected in two libraries of the green alga *Olltmansiellopsis viridis* (Fig. 1B). These may relate to contaminants that do not produce 18S sequences, as evidenced by the recent detection of Rhodobacteralean commensal sequences in 1kp libraries from *Mantoniella squamata* (QXSZ), *Bathycoccus prasinos* (MCPK) and *Nannochloropsis oculata* (JCFK) [19]. Additional results are provided in the associated data release.

Assembly qualities

We assessed the quality of each assembled scaffold using Transrate [20], which detects several classes of common assembly errors and assigns a quality score to each scaffold. Users of the data may choose to omit those portions of the assembly judged as low-quality when doing their own analyses.

Percentile	Good Contigs (all sizes)	Good Contigs - Percentage
5	19,355	32.47%
25	30,755	44.83%
50	37,983	53.65%
75	47,608	62.93%
95	71,368	74.87%

Completeness of gene set

Two different approaches were used to estimate transcriptome completeness. Firstly, BUSCO v1 [21] was applied with default settings, using the eukaryote and embryophyte conserved gene data sets (eukaryota_odb9, embryophyta_odb9) as the query databases. Secondly, conditional reciprocal best BLAST (CRBB) hits were calculated using CRB-BLAST [22] with default parameters. The predicted coding sequences were used as queries against the set of 248 core eukaryotic genes (CEGs) distributed with the CEGMA software (Core Eukaryotic Genes Mapping Approach); these 248 genes are highly conserved in eukaryotic genomes [23] and hence should be present in most transcriptomes.

As with all RNA-seq data, some genes are more highly expressed than others. While the CEGMA and BUSCO gene sets are intended to demonstrate the completeness of the transcriptomes, they are sensitive to the expression of these genes. Not all these genes will be expressed in the sample’s tissues

at sufficiently high levels to be assembled. A plot of the number of assembled scaffolds vs. the fraction of the three gene sets found in the assembled scaffolds shows an increase in the gene fractions found as the number of assembled scaffolds increases (Fig. 2). However, these quickly saturate at 80+% for the CEGMA and BUSCO-eukaryote sets, with a continuing rise over a larger range for the BUSCO-embryophyte set.

This shows that the three gene sets have somewhat different expression patterns, with the CEGMA and BUSCO-eukaryotic sets comprising genes that are more readily detected in our RNA samples. Some of the weaker sensitivity to the BUSCO-embryophyte set is attributable to our sampling species outside of this phylum, which may not have the homologous genes; however, the observed effect is larger than this and is also present when only the embryophyte samples are considered (not shown).

Percentage CEG abundance was calculated as number of CEGs with a CRBB hit divided by 248, the number of CEGs used. The percentage BUSCO abundance was calculated as 100% minus the missing percentage. Samples with low abundance by these measures should be treated with caution because the observed transcriptome incompleteness may indicate problems in library preparation or other types of poor sample quality. For these reasons the taxonomic analyses in Ref. 1 excluded samples with less than 57.5% BUSCO abundance. The table below shows the percentages of complete genes found for each of the three references at several percentile of the whole dataset.

Percentile	CEGMA 248	BUSCO – Embryophyta*	BUSCO – Eukaryota*
5	79.03	11.2 (8.5)	66.0 (37.3)
25	89.92	44.1 (29.8)	84.9 (64.4)
50	92.34	62.5 (48.2)	90.4 (75.9)
75	93.55	75.2 (59.6)	93.7 (84.1)
95	94.76	82.6 (73.2)	96.1 (91.0)

*Complete+fragment assemblies reported with complete sequences in parentheses.

Re-use potential

Since many of the samples are from poorly sequenced clades, the Thousand Plant sequence data is the first-large scale sequence data available for many species. We expect these sequences to be of broad interest to the plant sciences community, whether researchers merely use our sequences, supplement them with their own sequences, or develop PCR primer and probe sets to collect entirely new sequence data.

Availability of Supporting Data

Data to be in an associated *Gigascience*/GigaDB submission: [A copy of this is currently available at: https://drive.google.com/drive/folders/175nB8kf1UQushuEzv7UaJLPNNwdOrxh5?usp=sharing]

1. Tables with list of samples/assemblies (Sample-List-with-Taxonomy.tsv) and corresponding ENA/NCBI references (NCBI-ENA-Sequence-Identifiers.csv) and GigaDB links (to be added).

2. The major part of the provided data includes a FASTA files containing the SOAPdenovo-Trans assembly, the translation of the scaffolds to amino acids, the subset of the nucleotide sequence corresponding to the translation, and tab-separated (text) files with tables of Transrate outputs. These are available for each of the assemblies listed in the supplemental table. (onekp-data directory)

e.g. AALA-SOAPdenovo-Trans-assembly.fa.bz2, AALA-SOAPdenovo-Trans-translated.tar.bz2, AALA-

SOAPdenovo-Trans-Transrate-stats.tsv.gz, etc.

3. Two accessory tables containing details of the SILVA based SSU validation for each sample. The first (18S-analysis-Sample-Summary.xlsx) lists whether the sample is overall judged to be validated as containing the expected sequence, and whether it had alignments to any other plant sequences (described as worrisome contamination). The second file (18S-analysis-Scaffold-Results.xlsx), has more details listing each scaffold identified as being an 18S sequence, and which reference sequence it matched against.

4. The cross-contamination details. A summary file (Cross-contamination-Details.xlsx) includes a table (sheet Contamination Frequencies) with the number of contaminants, number of non-contaminant sequences, and the number of sequences inferred to be contaminants in other taxa for each sequence library.. Also included (sheet Contaminant Pairs) is a list of each pair of contaminant sequences identified, with the first column showing the contaminant sequence, and the second column the sequence corresponding to the orthologous contaminating partner against which the sequence was identified. Also included is a list of taxonomically close sample pairs which were not compared (sheet Excluded Taxa). Clean and contaminant FASTA sequence files for each library are available in the accompanying data (1kp_decontamination_libraries.gz.zip).

Declarations

The authors declare that they have no conflicting interests, and that they believe that all the plant tissues were collected in accordance with applicable regulations and laws.

References

1. One Thousand Plant Transcriptomes Initiative. A Phylogenomic View of Evolutionary Complexity in Green Plants. In review, 2019.

2. Wickett NJ, Mirarab S, Nguyen N, Warnow T, Carpenter E, Matasci N, Ayyampalayam S, Barker MS, Burleigh JG, Gitzendanner MA, Ruhfel BR, Wafula E, Der JP, Graham SW, Mathews S, Melkonian M, Soltis DE, Soltis PS, Miles NW, Rothfels CJ, Lisa Pokorny, Shaw AJ, DeGironimo L, Stevenson DW, Surek B, Villarreal J-C, Roure B, Philippe H, dePamphilis CW, Chen T, Deyholos MK, Baucom RS, Kutchan TM, Augustin MM, Wang J, Zhang Y, Tian Z, Yan Z, Wu X, Sun X, Wong GK-S, Leebens-Mack J. Phylotranscriptomic analysis of the origin and early diversification of land plants. Proc. Natl. Acad. Sci. USA 2014;111:E4859–E4868 doi:10.1073/pnas.1323926111

3. Johnson MTJ, Carpenter EJ, Tian Z, Bruskiewich R, Burris JN, Carrigan CT, Chase MW, Clarke ND, Covshoff S, dePamphilis CW, Edger PP, Goh F, Graham S, Greiner S, Hibberd JM, Jordon-Thaden I, Kutchan TM, Leebens-Mack J, Melkonian M, Miles N, Myburg H, Patterson J, Pires JC, Ralph P, Rolf M, Sage RF, Soltis D, Soltis P, Stevenson S, Stewart CN Jr, Surek B, Thomsen CJM, Villarreal JC, Wu X, Zhang Y, Deyholos MK, Wong GK-S. Evaluating Methods for Isolating Total RNA and Predicting the Success of Sequencing Phylogenetically Diverse Plant Transcriptomes. PLOS One 2012; doi:10.1371/journal.pone.0050226.

4. Jordon-Thaden IE, Chanderbali AS, Gitzendanner MA, Soltis DE. Modified CTAB and TRIzol Protocols Improve RNA Extraction from Chemically Complex Embryophyta. Appl in Plant Sci 2015;3:1400105 doi:10.3732/apps.1400105.

5. Mueller O, Lightfoot S, Schroeder A. Agilent Technologies Application Note: RNA Integrity Number (RIN) – Standardization of RNA Quality Control. 2016. https://www.agilent.com/cs/library/applications/5989-1165EN.pdf

6. Xie Y, Wu G, Tang J, Luo R, Patterson J, Liu S, Huang W, He G, Gu S, Zhou SLX, Lam T-W, Li Y, Xu X, Wong GK-S, Wang J. SOAPdenovo-Trans: de novo transcriptome assembly with short RNA-Seq reads. Bioinformatics 2014;30:1660–1666 doi:10.1093/bioinformatics/btu077.

7. Barker MS, Dlugosch KM, Dinh L, Challa RS, Kane NC, King MG, Rieseberg LH. EvoPipes.net: Bioinformatic tools for ecological and evolutionary genomics. Evol. Bioinfo. 2010;6:143–149 doi:10.4137/EBO.S5861.

8. Goodstein DM, Shu S, Howson R, Neupane R, Hayes RD, Fazo J, Mitros T, Dirks W, Hellsten U, Putnam N, Rokhsar DS. Phytozome: a comparative platform for green plant genomics. Nucl. Acids Res. 2012;40:D1178–D1186 doi:10.1093/nar/gkr944.

9. Wheeler DL, Barrett T, Benson DA, Bryant SH, Canese K, Chetvernin V, Church DM, Dicuccio M, Edgar R, Federhen S, Feolo M, Geer LY, Helmberg W, Kapustin Y, Khovayko O, Landsman D, Lipman DJ, Madden TL, Maglott DR, Miller V, Ostell J, Pruitt KD, Schuler GD, Shumway M, Sequeira E, Sherry ST, Sirotkin K, Souvorov A, Starchenko G, Tatusov RL, Tatusova TA, Wagner L, Yaschenko E. Database resources of the National Center for Biotechnology Information. Nucl. Acids Res. 2008;36:D13–D21 doi:10.1093/nar/gkm1000.

10. Birney E, Clamp M, Durbin R. GeneWise and Genomewise. Genome Res. 2004;14:988–995 doi:10.1101/gr.1865504.

11. Camacho C, Coulouris G, Avagyan V, Ma N, Papadopoulos J, Bealer K, Madden TL. BLAST+: architecture and applications. BMC Bioinformatics 2009;10:421. doi:10.1186/1471-2105-10-421.

12. Lusk RW. Divese and Widespread Contamination Evident in the Unmpped Depths of High Throughput Sequencing Data. PLoS ONE 2014;9(10) e110808 doi:10.1371/journal.pone.0110808.

13. Quast C, Pruesse E, Yilmaz P, Gerken J, Schweer T, Yarza P, Peplies J, Glöckner FO. The SILVA ribosomal RNA gene database project: improved data processing and web-based tools. Nucl. Acids Res. 2013;41:D590–D596 doi:10.1093/nar/gks1219.

14. Dorrell RG, Gile G, McCallum G, Méheust R, Bapteste EP, Klinger CM, Brillet-Guéguen L, Freeman KD, Richter DJ, Bowler C. Chimeric origins of ochrophytes and haptophytes revealed through an ancient plastid proteome. Elife 2007; 6, 23717 doi:10.7554/eLife.23717.

15. Dorrell RG, et al. (2019) Contrasting evolutionary fates accompany the loss of photosynthesis in different heterotrophic chrysophytes. Proc Natl Acad Sci USA, in press.

16. Marron AO, Ratcliffe S, Wheeler GL, Goldstein RE, King N, Not F, de Vargas C, Richter DJ. The Evolution of Silicon Transport in Eukaryotes. Mol Biol Evol 2016;33(12):3226-3248 doi:10.1093/molbev/msw209.

17. Kiełbasa SM, Wan R, Sato K, Horton P, Frith MC. Adaptive seeds tame genomic sequence comparison. Genom Res 2011;21(3):487-493 doi:10.1101/gr.113985.110.

18. Moreno-Hagelsieb G, Latimer K. Choosing BLAST options for better detection of orthologs as reciprocal best hits. Bioinformatics 2008;24(3):319-324 doi:10.1093/bioinformatics/btm585.

19. Sato S, Nanjappa D, Dorrell RG, Jimenez Vieira FR, Kazamia E, Tirichine L, Veluchamy A, Jaillon O, Wincker P, Fussy Z, Kuo A, Obornik M, Munoz-Gomez SA, Mann DG, Bowler C, Zingone A.

Genome-enabled phylogenetic and functional reconstruction of an araphid pennate diatom CCMP470, previously assigned as a radial centric diatom, and its bacterial commensal. Manuscript submitted.

20. Smith-Unna R, Boursnell C, Patro R, Hibberd J, Kelly S. TransRate: reference free quality assessment of de novo transcriptome assemblies. Genome Res. 2016;26:1134–1144; doi:10.1101/gr.196469.115.

21. Simão FA, Waterhouse RM, Ioannidis P, Kriventseva EV, Zdobnov EM. BUSCO: assessing genome assembly and annotation completeness with single-copy orthologs. Bioinformatics 2015;31:3210–3212 doi:10.1093/bioinformatics/btv351.

22. Aubry S, Kelly S, Kümpers BMC, Smith-Unna RD, Hibberd JM. Deep Evolutionary Comparison of Gene Expression Identifies Parallel Recruitment of Trans-Factors in Two Independent Origins of C4 Photosynthesis. PLOS Genetics 2014 doi:10.1371/journal.pgen.1004365.

23. Parra G, Bradnam K, Ning Z, Keane T, Korf I. Assessing the gene space in draft genomes. Nucl. Acids Res. 2009;37:289–297 doi:10.1093/nar/gkn916.

Figure Captions:

Fig. 1. Panel A provides an overview of the total sequence percentage verified to be of contaminant origin (red), or inferred to be possible contaminants in other sequence libraries (grey) in all 1kp libraries, and libraries inferred to be contaminated through other techniques (e.g. 18S phylogenetic placement). Panel B lists 21 libraries in which > 6% of the total sequences are potential contaminants. Panel C shows a heatmap of inferred contaminant interactions between pairs of species; contaminated species are shown on the vertical axis, and contaminating species on the horizontal axis.

Fig. 2. Fraction of the gene sets found (complete + fragments) versus the number of scaffolds (300+ bp) in the assemblies. For each sample, the fraction of the eukaryota and embryophyta sets found in the assemblies are calculated with BUSCO and the fraction of the CEGMA 248 set with the CRBB tool. All three sets are more completely recovered at higher scaffold counts, but the BUSCO embryophyta set is less complete in our samples.

Figure 1

Click here to download Figure Fig-1.pptx

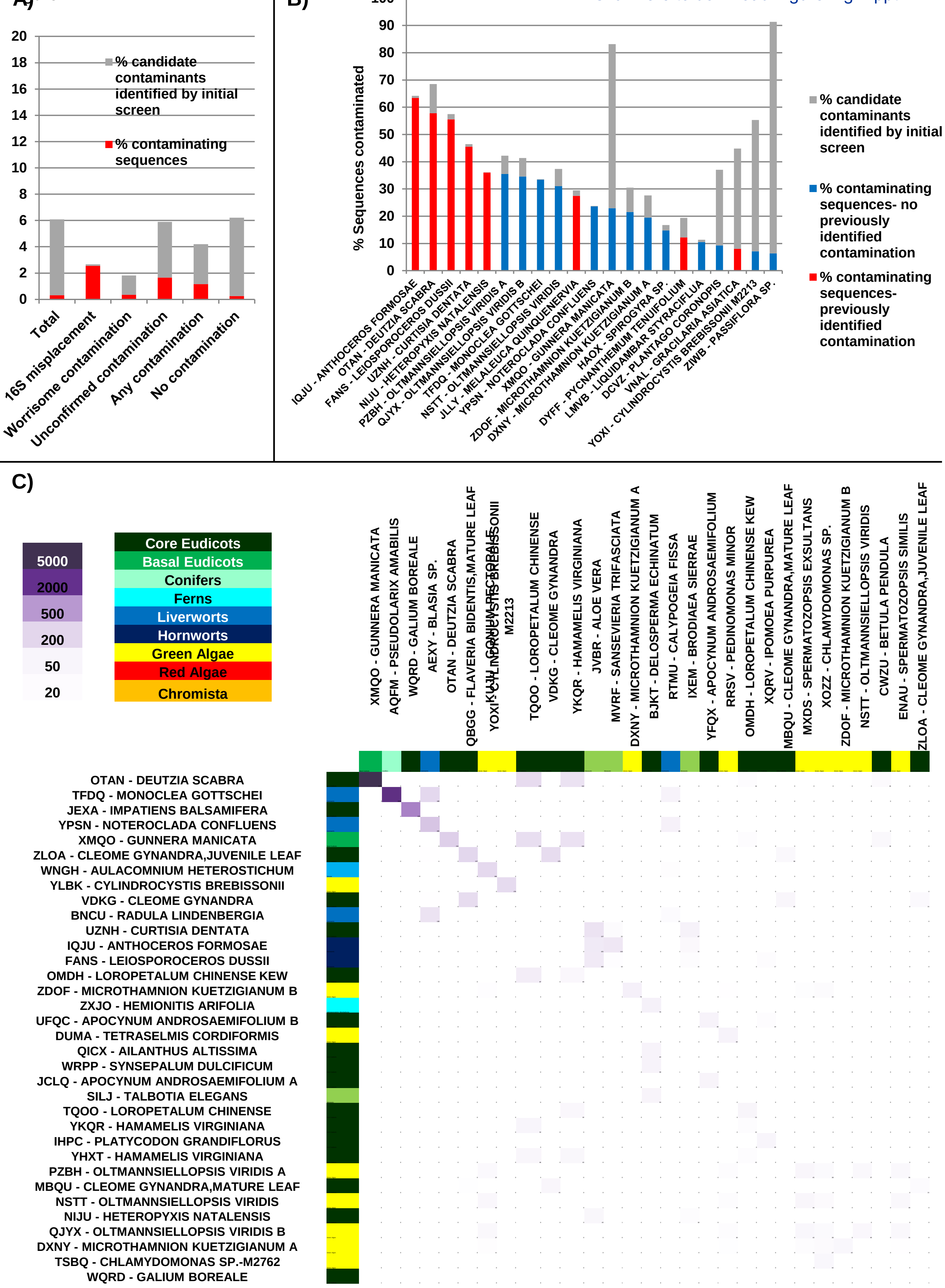

Figure 2

Click here to download Figure Fig-2.png

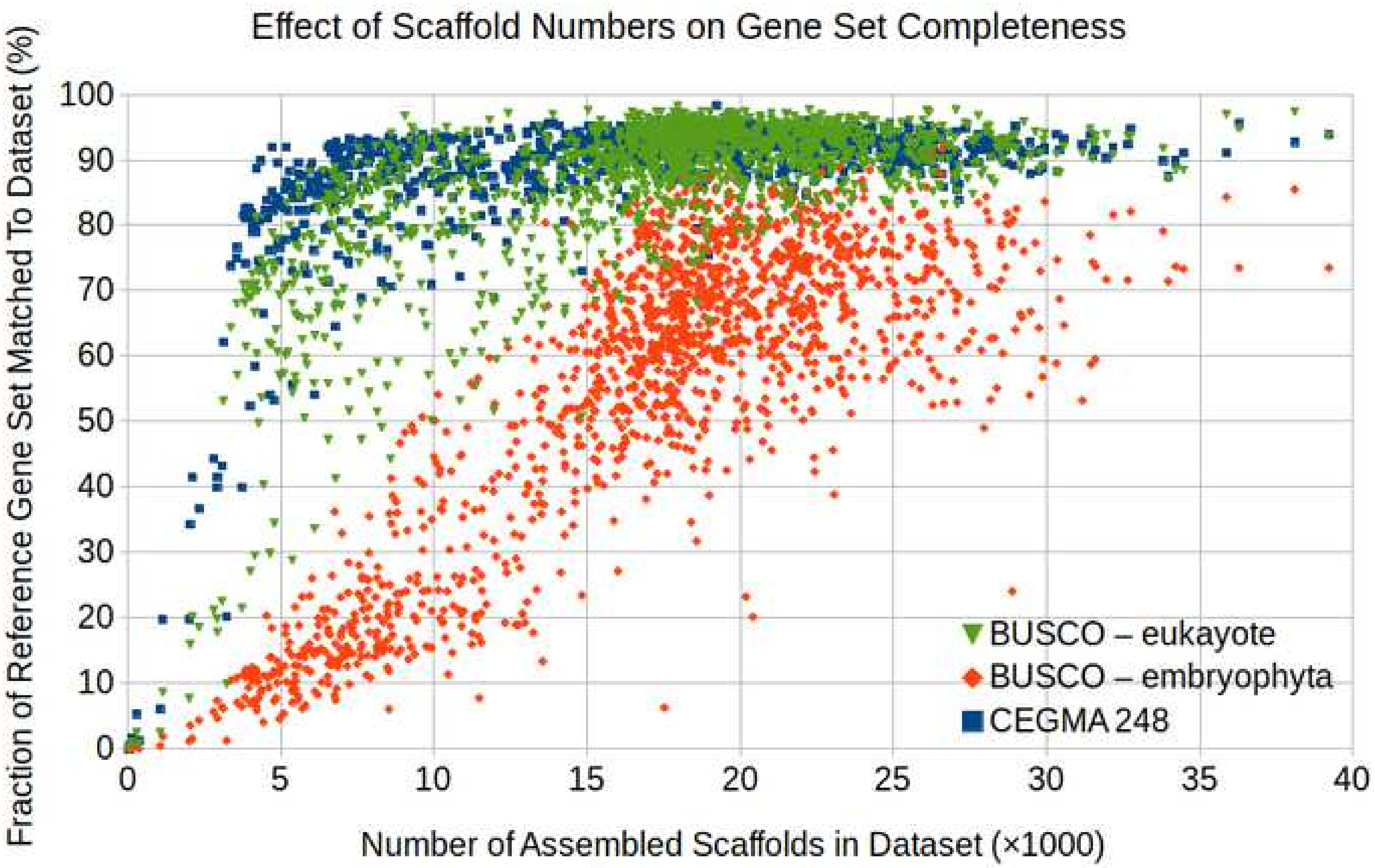

Supplement: giz126_GIGA-D-19-00241_Original_Submission [file giz126_giga-d-19-00241_original_submission.pdf]
