# Supplementary material for: Access to RNA-sequencing data from 1,173 plant species: The 1000 Plant transcriptomes initiative (1KP)
Source: Gigascience. 2019 Oct 23;8(10):giz126. doi: 10.1093/gigascience/giz126 (PMC6808545; doi:10.1093/gigascience/giz126)
Supplement: giz126_Response_to_Reviewer_Comments_Original_Submission [file giz126_response_to_reviewer_comments_original_submission.pdf]

Author's Response To Reviewer Comments

Close

Response to Reviewers Comments:

Reviewer #1:

1, phylogenomic analyses need alignments of orthologous genes, but this data note didn't provide them. Can this dataset be used in phylogenomic analysis?

This paper is a companion to Ref. 1, which deals with the phylogenomic analyses. Specifics of the phylogenomic analysis including the process of generating alignments between orthologous genes are more properly discussed in Ref. 1 and it's online supplements and are not discussed here.

2, please explain the tables in Line109, 282 and 317.

all three tables now have a title and legend and are referenced from the body text

3, for many species selected here, their transcriptomes had been sequenced before. Why don't use these pre-existing data? How to determine the superiority of the data provided in this paper?

This paper describes a data set that was generated some time ago, primarily for a complex phylogenomics analysis just accepted for publication in a major journal. We are not claiming our data is the best available for any given species. Although we tried to avoid overt duplication, considering the time involved, it should come as no surprise that other groups may have also sequenced the same species.

4, evolutionary complexity includes many aspects, including variation in chromosomal structures and the numbers. Can the transcriptomic sequences capture the substantial phylogenomic signals of so many plants? Why?

Again, the phylogenomics was reviewed in detail for the capstone paper. This is just a paper to describe the data set used.

Reviewer #2

-Table legends are missing and needs to be added. Also be consistent using "th" percentile throughout the three tables.

legends have been added and the th suffix is used in all three tables

-line 112: quantify "excessive", level of reads removed?

"excessive" is not the correct word and has been removed for clarity. We do not have data on how extensive this removal was. We expect that it should have been only a small fraction of the total reads sequenced.

-line 125: what is the dataset AEPI?

Discussion of the dataset ID codes used has been added. Dataset AEPI was selected as an example.

-line 142 [cite]?
A placeholder reference to the protocols.io entry is now present.

-line 146 I find the title "Protein translation" a bit strange since it is prediction of coding regions it refers to

This title has been adjusted to better match the material.

-line 153 "those" what?

those codons - text changed

-line 154 Sentence "Outputs…" remove or point at where the output files are

reference to the associated data added

-line 165 and 166: maybe a miss something but the nucleotide sequences are 1/5 of the predicted protein sequences after Transpipe…?

This is correct. Some description has been added to the previous paragraph to help emphasize that the process only translates a portion of the material. (Those assemblies with sufficient similarity the the Phytozome reference sequences.)

-line 175: this statement needs a reference

No reference is available. We have removed the comment.

-line 193: "these problems", please be more specific, and clearly list which ways tried

The other methods are ad hoc analyses and are not as universally applicable as the 18S based analyses. We do not want to waste time/space with detailed discussion of them. The text is rewritten to remove the references to them.

-line 237: can't access github page!

GitHub has been contacted about this and the issue seems to be fixed.

-line 307-308: I don't follow the last part of the argument as BUSCO - Emboryophyta looks fairly linear to number of assembled scaffolds and non-phylum samples should fall outside this linearity (which they might - can they be marked in any way)

After consideration the wording has been changed to make the weaker statement that the difference remains if only the embryophyte samples are considered.

In fig 1 panel B: what are the 4-letter abbreviation before the species ntainingi names?

Discussion of the 4-letter codes has been added to the main text of the paper.

Close
