# Supplementary material for: Access to RNA-sequencing data from 1,173 plant species: The 1000 Plant transcriptomes initiative (1KP)
Source: Gigascience. 2019 Oct 23;8(10):giz126. doi: 10.1093/gigascience/giz126 (PMC6808545; doi:10.1093/gigascience/giz126)
Supplement: giz126_Response_to_Reviewer_Comments_Revision_1 [file giz126_response_to_reviewer_comments_revision_1.pdf]

Author's Response To Reviewer Comments

Close

Final revisions made as requested.

Close
