# Supplementary material for: Access to RNA-sequencing data from 1,173 plant species: The 1000 Plant transcriptomes initiative (1KP)
Source: Gigascience. 2019 Oct 23;8(10):giz126. doi: 10.1093/gigascience/giz126 (PMC6808545; doi:10.1093/gigascience/giz126)
Supplement: giz126_Reviewer_2_Report_Original_Submission — Erik Alexandersson -- 7/6/2019 Reviewed [file giz126_reviewer_2_report_original_submission.pdf]

Reviewer Report

Title: Access to RNA seq data from 1,173 green plant species: the 1000 Plant Transcriptomes Initiative (1KP)

Version: Original Submission **Date:** 7/6/2019

Reviewer name: Erik Alexandersson

Reviewer Comments to Author:

The paper by Carpenter et al gives background information on the construction of the de novo transcriptomes assemblies for the 1000 plants initiative. Focus is on assembly completeness and quality as well as dealing with contaminants. This is a very important initiative for the plant community which findings and impact will go beyond internal use for researchers.
As a this project has been a communal effort it has of course taken long - it started in 2012 - to reach the end-goal and this is reflected in that development in sequencing standards/possibilities have advanced over time. Today, combinations of platforms for long and short read-lengths would have been considered. The authors have stuck to completeness of transcriptome, and for example no N50-values are given, to judge assembly quality. I think this is sufficient. However, was any action taken (removal) for transcriptomes with low completeness? The second half of the paper is dedicated to the identification of contaminants which is an interesting topic.
The paper should be published but there are some room for improvement and the results can be presented in a more stringent way.
Specific comments
-Table legends are missing and needs to be added. Also be consistent using "th" percentile throughout the three tables. Please also go through figure legends and improve the information content.
-line 112: quantify "excessive", level of reads removed?
-line 125: what is the dataset AEPI?
-line 142 [cite]?
-line 146 I find the title "Protein translation" a bit strange since it is prediction of coding regions it refers to
-line 153 "those" what?
-line 154 Sentence "Outputs…" remove or point at where the output files are
-line 165 and 166: maybe a miss something but the nucleotide sequences are 1/5 of the predicted protein sequences after Transpipe…?
-line 175: this statement needs a reference
-line 193: "these problems", please be more specific, and clearly list which ways tried
-line 237: can't access github page!
-line 307-308: I don't follow the last part of the argument as BUSCO - Emboryophyta looks fairly linear to number of assembled scaffolds and non-phylum samples should fall outside this linearity (which they might - can they be marked in any way)

In fig 1 panel B: what are the 4-letter abbreviation before the species names?

Level of Interest

Please indicate how interesting you found the manuscript: Choose an item.

Quality of Written English

Please indicate the quality of language in the manuscript: Choose an item.

Declaration of Competing Interests

Please complete a declaration of competing interests, considering the following questions:

- Have you in the past five years received reimbursements, fees, funding, or salary from an organisation that may in any way gain or lose financially from the publication of this manuscript, either now or in the future?
- Do you hold any stocks or shares in an organisation that may in any way gain or lose financially from the publication of this manuscript, either now or in the future?
- Do you hold or are you currently applying for any patents relating to the content of the manuscript?
- Have you received reimbursements, fees, funding, or salary from an organization that holds or has applied for patents relating to the content of the manuscript?
- Do you have any other financial competing interests?
- Do you have any non-financial competing interests in relation to this paper?

If you can answer no to all of the above, write 'I declare that I have no competing interests' below. If your reply is yes to any, please give details below.

I declare that I have no competing interests

I agree to the open peer review policy of the journal. I understand that my name will be included on my report to the authors and, if the manuscript is accepted for publication, my named report including any attachments I upload will be posted on the website along with the authors' responses. I agree for my report to be made available under an Open Access Creative Commons CC-BY license (http://creativecommons.org/licenses/by/4.0/). I understand that any comments which I do not wish to be included in my named report can be included as confidential comments to the editors, which will not be published.

Choose an item.

To further support our reviewers, we have joined with Publons, where you can gain additional credit to further highlight your hard work (see: https://publons.com/journal/530/gigascience). On publication of this paper, your review will be automatically added to Publons, you can then choose whether or not to claim your Publons credit. I understand this statement.

Yes Choose an item.
